# Environmental Exposures and Child Health: What we Might Learn in the 21st Century from the National Children’s Study?

**DOI:** 10.4137/EHI.S1061

**Published:** 2008-11-06

**Authors:** Jane A. McElroy

**Affiliations:** Assistant Professor, University of Missouri, Family and Community Medicine, MA306 Medical Science Building, Columbia, MO 65212

**Keywords:** child health, longitudinal study, environmental exposures, epidemiology

In utero and early life environmental exposure programming may be critical to the onset of many diseases and dysfunctions in adulthood (Barker, 2007; Heindel, 2006), such as an increased risk of hypertension, cardiovascular disease (Palinski et al. 2007), diabetes (Armitage et al. 2008), and breast cancer (Xue and Michels, 2007). Some in utero and early life environmental exposures have demonstrated unequivocal harmful effects to children, such as thalidomide (Speirs, 1962), ionizing radiation (Doll and Wakeford, 1997), methylmercury (Watanabe and Satoh, 1996), lead (Gardella, 2001), environmental tobacco smoke (Herrmann et al. 2008), cigarette smoking and high alcohol consumption during pregnancy (Krulewitch, 2005; Shea and Steiner, 2008). Whereas other in utero and early life environmental exposures have come under scrutiny without consensus as to the degree of influence on child or adult health, such as exposure to pesticides (Eskenazi et al. 1999), polybrominated biphenyls (PCBs) (Guo et al. 1995), nitrates (Mueller et al. 2004), bisphenol A (vom Saal and Hughes, 2005), and phytoestrogens (Damgaard et al. 2002).

Several factors converge to make susceptibility to adverse effects of environmental exposures of particular importance during the developmental periods of the fetus and child. These include host vulnerability during organogenesis and neuronal development; lower exposure thresholds and metabolic capacity of children relative to adults; behavioral factors that may increase children’s ingestion of environmental toxins (Goldman et al. 2004); and inhalation of harmful air pollutants (Bates, 1995). Furthermore, during fetal and infant development, vulnerability to some exposures is increased during critical windows (Selevan et al. 2000). Attempts to tease out the timing of exposure associated with health outcomes are challenging. Unfortunately, our understanding of the importance of common environmental exposures occurring in utero and early life with regard to influence on child and adult health has been slow. For example, one misconception that took considerable time to alter was the belief that the fetus was protected from exposures—the placenta barrier misconception (Welshons et al. 2003). With this paradigm shift, we now realize that the fetus may face significant environmental exposures and in some cases a biomagnification of exposure (Ask et al. 2002; Hanrahan et al. 2004). Further, bioaccumulation of some chemicals, such as persistent organic pollutants (POPs) in children are higher than in their parents (Thundiyil et al. 2007; Trapp et al. 2008). This has lead to an active research area investigating epigenetic changes occurring from in utero exposures which may affect child or adult health (Dolinoy et al. 2007). Much has been learned about an environmental stressor of the Dutch winter famine of 1944–1945 and specific long-term consequences of this deprivation on fetuses (Ravelli et al. 1998; Ravelli et al. 1999), which may be due to epigenetic changes of the DNA (Jablonka et al. 1992). Ibanez et al. suggested that female infants born small for gestational age may have subfertility (Ibanez et al. 2002). Another area of active research is identifying and quantifying the factors associated with the well-documented disproportional burden of illness, disease, and mortality among certain groups (Haas, 2008; Larson et al. 2008).

Some of what we have learned about in utero exposure has been due to obvious heath outcomes. For example, the synthetic form of estrogen, diethylstilbestrol (DES) was prescribed to pregnant women to prevent miscarriages from 1938–1971 (Gunning, 1976). In 1970, Herbst and colleagues reported on seven young women presenting at their hospital of an extremely rare vaginal cancer; a cancer never before seen at that hospital in that age group (Herbst et al. 1971). This began the investigation and subsequent conclusive identification of DES as the culprit. Similarly, a dramatic increase in number of children borne with severe malformities, including phocomelia, led to discovering the thalidomide link, a medication prescribed to pregnant mothers as an antiemetic to combat morning sickness and as a sleep aide (Knightley et al. 1979).

The patterns of illness and disease among U.S. children have significantly changed over time. Chronic conditions, such as learning disabilities, attention deficit/hyperactivity disorder, premature birth, asthma, obesity, autism, and type 2 diabetes, are more likely to be experienced by children today than by children born in the early 20th century (Schor, 2007). Similarly, the environment in which fathers, mothers and children live has also substantially changed (Goldman, 1998). Noteworthy improvements have occurred in assistive reproductive technologies, prenatal screening, and neonatal care during the 20th century (Budak et al. 2007; Wren et al. 2008). In contrast, consistent findings have shown that children walk less, spend more time interacting with computers, particularly gaming, eat out more, consume more sugared drinks which has led to concern about the health effects of these changes (Centers for Disease Control and Prevention (CDC) 2005; Demory-Luce, 2005; Larson, 2001; Wang et al. 2008). Further, children have higher prevalence of medication use and vaccine compliance though the benefit/risk of the use of medications and immunization is currently passionately debated among certain groups (Berkowitz et al. 2001). With regard to chemical exposure, this too has changed over time. The 1950’s ushered in the “better living through chemicals” age and this trend in use of synthetic chemicals, such as flame retardants, pesticides, plastics, chemotherapeutic agents, and building materials, continues to grow with now over 80,000 synthetic chemicals in use (U.S. Environmental Protection Agency 2007). Over one million tons of approximately 3,000 chemicals, called high production volume chemicals, are produced each year (U.S. Environmental Protection Agency 2008). These synthetic chemicals are widely dispersed in air, water, soil, foods and consumer products. National surveys of the U.S. population have detected synthetic chemicals in biospecimen samples from children as well as adults (National Health and Nutrition Examination Survey (U.S.) and National Center for Environmental Health (U.S.), Division of Laboratory Sciences 2005). Another facet of the changing landscape is the shift from a life in the country to one in the city or suburbs with all the lifestyle changes that accompany living in an (sub)urban environment (U.S. Census Bureau, 2008). The built environment encompasses a broad range of definitions from housing stock’s age, construction, and building materials to the local neighborhood’s land-use (Cummins and Jackson, 2001). Many aspects of the built environment affect children’s health risk such as easy access to recreational facilities, greater walkability, interconnectivity of neighborhoods, sidewalks, density of fast food restaurants and convenience stores, etc (Grafova, 2008). In addition, changes in building construction have been reported to influence children’s health, such as increased air tightness of homes, and building materials (Breysse et al. 2004; Sherman and Matson, 2002). Those that live in low-income neighborhoods are particularly at risk (Gordon-Larsen et al. 2006). Although the magnitude of influence of specific factors that lead to this increased risk has yet to be elucidated, disparities in child and adult health have been well documented (Northridge et al. 2003).

Due to the dramatic changes in our environment, our advances in efficiently quantifying biomarkers of exposure, and the rise in chronic health conditions facing today’s children, the U.S. Congress demonstrated support for research focusing on causes of children’s illnesses and diseases. In 2000, the U.S. Congress called for “a national longitudinal study of environmental influences on children’s health and development” by enacting the Children’s Health Act of 2000 (Children’s Health Act of 2000. Public Law 106-310 2000). The Children’s Health Act of 2000 authorized the National Institute of Child Health and Human Development as well as a consortium of federal agencies including, the Centers for Disease Control and Prevention, the National Institute of Environmental Health Sciences, and the U.S. Environmental Protection Agency, with input from working teams of researchers throughout the country, to design a study, including compiling a list of relevant hypotheses, to address children’s health and development (Kimmel et al. 2005; Trasande and Landrigan, 2004); (http://www.nationalchildrensstudy.gov/about/partners/contributors/Pages/default.aspx lists many of the key people and institutions who contributed time and expertise to the development of this study and its hypotheses).

In brief, the goal of the National Children’s Study (NCS) is to establish a cohort of 100,000 children. The study will follow children and families for 21 years while collecting health information at different ages and stages of child development as well as in various situations. The prospective data collected at preconception through adulthood may be able to elucidate factors that influence health and development as children grow. A national multistage stratified probability sample that selected 105 study locations (areas/neighborhoods within selected counties) was chosen for the recruitment of participants (see http://www.nationalchildrensstudy.gov/studylocations/Pages/map.aspx for map of the locations of the study locations). The plan is to enroll and obtain the first biospecimens from 25% of the women pre-conception, 65% of the women before their second trimester and the remaining 10% of the women at the birth event. The NCS hypotheses are categorized into seven broad groups 1) pregnancy outcomes, 2) neurodevelopmental and behavior, 3) child health and development, 4) asthma, 5) obesity and growth, 6) injury and 7) reproductive development. Under each group are one to six hypotheses. For example, under reproductive development, one hypothesis is “prenatal and postnatal (including peripubertal) exposure to hormonally active environmental agents can alter development of the reproductive system resulting in multiple types of outcomes that can occur at various stages of development and may result in cumulative effects over time.” (http://www.nationalchildrensstudy.gov/research/hypotheses/Pages/hypotheses_list.aspx). Bio-markers and survey data will be gathered at numerous time points during the study (see http://www.nationalchildrensstudy.gov/research/hypotheses/Pages/hypotheses_list.aspx for a version of the protocol overview and summary of contacts for data collection, through month 24 under appendix H).

At the time of writing (September 2008), the study is waiting for the OMB (Office of Management and Budget) to approve the study. Once this approval and local Institutional Review Board (IRB) approvals are obtained, contact with study participants in the Vanguard Centers (centers that will collect one year of pilot data) can begin. In 2005, seven Vanguard centers and the Coordinating Center was funded; in 2007 Congress appropriated $69 million for this study and an additional 22 study centers were awarded; in 2008, Congress appropriated $110.9 million with approximately half of the remaining study centers to be awarded in September, according to the March 19th, 2008 Request for Proposal (see http://www.nationalchildrensstudy.gov/about/pages/funding.aspx for the yearly appropriations since the onset in 2000). The NCS plan is to have study centers identified for all 105 study locations by 2010. (http://www.nationalchildrensstudy.gov/research/hypotheses/Pages/hypotheses_list.aspx for list of counties by funded status). Needless to say, without substantial yearly financial support by the federal government, the study will not be able to continue as it is currently designed.

In summary, current advances in genomics, identification of relevant biomarkers, and statistical analysis of complex data will enable researchers to evaluate effect modification and interaction of multiple genetic and environmental factors in the etiology of childhood illnesses, injuries and disease.

Parents confronted with sick children often wonder whether an environmental exposure was the culprit of the child’s health problem. Unfortunately, strong evidence of environmental exposures associated with health outcomes is rarely available. Similar to the Framingham Heart Study which provided critical information on preventable risk factors for cardiovascular disease in adults that changed many facets of heart care (http://www.nhlbi.nih.gov/about/framingham/), findings from cohort studies, such as the National Children’s Study, holds a similar promise for understanding the influence of environmental exposures associated with child and adult health.
